# Usability survey of biomedical question answering systems

**DOI:** 10.1186/1479-7364-6-17

**Published:** 2012-09-01

**Authors:** Michael A Bauer, Daniel Berleant

**Affiliations:** 1 Department of Information Science, University of Arkansas at Little Rock, 2801 S. University Ave, Little Rock, AR, 72204, USA; 2 University of Arkansas for Medical Sciences, 4301 W. Markham St., Little Rock, AR, 72205, USA

## Abstract

We live in an age of access to more information than ever before. This can be a double-edged sword. Increased access to information allows for more informed and empowered researchers, while information overload becomes an increasingly serious risk. Thus, there is a need for intelligent information retrieval systems that can summarize relevant and reliable textual sources to satisfy a user's query. Question answering is a specialized type of information retrieval with the aim of returning precise short answers to queries posed as natural language questions. We present a review and comparison of three biomedical question answering systems: askHERMES (http://www.askhermes.org/), EAGLi (http://eagl.unige.ch/EAGLi/), and HONQA (http://services.hon.ch/cgi-bin/QA10/qa.pl).

## Introduction

There are numerous general purpose search engines available online, but as information sources continue to proliferate, specialized and domain-specific information retrieval tools become more essential. One such domain is the clinical and biomedical fields, where the body of scientific knowledge is large and increasing. To minimize searching and browsing time while maximizing usefulness of that knowledge and data, we are seeing considerable interest in biomedical/clinical question answering systems [1]. Question answering (QA) is a specialized type of information retrieval that returns precise short answers to queries posed as natural language questions [2-5]. It is the goal of such systems to move the burden of skimming multiple documents, which can be quite time consuming, from the researcher or clinician to the computer. The recent successes of IBM's Watson on Jeopardy highlight the possibilities and potential power of QA [6]. We present a review of three leading biomedical QA systems, askHERMES [7-9], EAGLi [10,11], and HONQA [12-14], which are all publically accessible online. This paper is organized into sections based on key usability dimensions used to compare the different systems.

## Information sources

An important factor for any domain-specific QA system is the accuracy and trustworthiness of the sources against which queries are performed. Most biomedical QA systems make use of MEDLINE abstracts as an information source [15]. Two systems that we reviewed, askHERMES and EAGLi, used MEDLINE as a major source of answers. In addition, askHERMES uses eMedicine, [16] clinical guidelines, PubMedCentral [17] full text documents, and Wikipedia. EAGLi uses Medical Subject Headings to help answer some definitional questions. HONQA uses websites that have been certified by Health On the Net Foundation (HON) [18], unlike the other two systems that rely heavily on MEDLINE.

## Response time and results

First of all, the systems vary in their response times and in the form of answers returned to the user (in particular, single or multiple sentences). All three QA systems return relatively short answers to clinical or biomedical questions instead of entire documents. Response time assessment is based on the relative amount of time it took each system to respond to a typical query.

EAGLi is quite slow and may not truly be ready for high volume traffic. In response to a question that the system ‘understands,’ a list of possible answers is displayed with corresponding levels of confidence indicated. Links to abstracts are also provided and grouped by which answers to the question they support. If a question is not understood, EAGLi returns a list of abstracts that contained some of the query terms. The program also provides a short snippet of text from the abstract that contains keywords from the query. Next to the text there are links to PubMed and to a page they call a ‘semantic summary’ which displays the entire abstract and a list of all the Gene Ontology and SwissProt terms that were matched, along with the phrase they were mapped to. A score is given to indicate to the user the strength of the mapping. This information gives the user a way to understand why the system has determined that a particular abstract supports an answer or was given as the answer. A link to a matrix is provided on the main results page that can quickly give the user an overview of the terms that were matched in the abstracts. This system provides a degree of transparency to the retrieval process that traditional information retrieval systems hide from the user. That in turn supports efforts by the user to efficiently figure out how to best phrase a query or question to get the most relevant information.

The askHeremes system responds significantly more quickly than EAGLi or HONQA. It warns that it may take up to 60 s, but more often than not, it returns results in only a few seconds. Query terms are determined first by identifying noun phrases in a question which are then weighted based on several methods. The query is subsequently expanded using the Unified Medical Language System (UMLS), dictionaries, and thesauruses. Answers that are returned in response to a question can be viewed in three different arrangements: clustered answers, ranked answers, and content clustered answers. Clustered answers are first grouped according to different combinations of query and UMLS query expansion terms. They are then sub-clustered by different combinations of synonym concepts. This functionality can be useful in answering a complex question, such as one about a cause and treatment, which may require reading several different passages to find an answer. This is useful because often a sufficient answer cannot be found in just one sentence or short passage. Content clustered answers provide a third method to view answers. Common labels are found for the original clusters, and additional answer passages are found that match these labels. This approach allows a passage to be found under multiple, easy to read labels. A list of related questions is shown and can be used to further refine the one's own query question. The answers returned by the system are short passages or phrases from MEDLINE abstracts which are linked back to the original citation. The system classifies questions into several categories defined by the National Library of Medicine (NLM) [19], such as diagnosis, treatment and prevention, etiology, pharmacological, management, and others. This classification aids in identifying query terms to use in retrieval.

HONQA is about as slow as EAGLi but it does display a status bar so that you can better tell whether it is working or has hung. Next to each answer, you can indicate whether a response to the question was appropriate or not. This is intended to help improve the quality of the answers provided by the system over time. Answers are linked to cached versions of the websites from which the sentences were obtained. The answers are sentences taken from HON certified websites. A health and medical website can apply to be certified, after which the HON organization will evaluate the site to see that it meets ‘The HON Code of Conduct for medical and health Web sites’ (HONcode) [20]. The use of certified health websites as a source of knowledge is unique to the HONQA system. It was the intent of the designers of HONQA that users with different levels of health and biological knowledge be able to benefit from answers that are understandable and useful. MEDLINE contains high quality peer reviewed literature but can be technically difficult to understand, whereas websites are typically designed and geared for a more diverse audience. However, a significant problem with using the Internet as a source of health information is the lack of oversight of the information that is presented. The HON certification helps alleviate the problem of incorrect and possibly dangerous medical information on the Internet. Another benefit of using websites as a knowledge source is that there are links to additional information present in most web pages (and absent from MEDLINE abstracts) that can often help answer the question if the sentence returned does not completely answer it.

## User interface

EAGLi provides a simple and clean interface which allows users to ask a question and either use the PubMed search tool or their specialized relevance driven search engine. Most of the items on the page can be hovered over with the mouse to display a small tooltip containing a more detailed description of the item. The terms that are selected from the question to be used to query are displayed on the results page. The system appears to reformulate and automatically expand the queries with the addition of Gene Ontology and SwissProt terms.

The interface to askHEREMES is also simple and clean with multiple tabs. At the top of the results page are links to clinical question answering tools, which include utilities to browse questions, classify question, and generate query terms. A question browsing utility allows browsing the NLM collection of clinical questions that they used while developing and tuning the system. A question classifying utility lets the user submit a question and see in which category the question is categorized. An *ad hoc* question can also be submitted to the query term generating utility to get a list of the keywords that would be extracted and used by the system to query the database. These utilities can help the user understand how the system answers questions that are posed, similar to the ‘Semantic Summary’ of EAGLi.

HONQA has a very simple and easy-to-understand interface. When results are returned, information about how the question was interpreted is provided and includes: the number of answers, the language, expected question type, and expected medical type. HONQA does some interpretation of the question to determine the type and kind of medical information being requested. Question types can be definition, factoid, list, and Boolean. The medical types a question may be include definition, diagnostic, physiology, and treatment. This helps the user determine if the system understands the intent of their question.

## Answer quality

The askHERMES system returns passages that could potentially answer all types of questions. A drawback is the consequently high recall; a large number of results are often returned, which tends to defeat the intent of a question answering system in reducing the amount of information that must be read. HONQA returned fewer answers to many biomedical questions and is tuned for medical questions. We observed that HONQA was able to present sentences that answered questions to definitional clinical questions. The sentences returned by the system were clear and easy to understand, and often, following links to the cached source texts for further elaboration was unnecessary. The EAGLi system was unique in that, when it understood a definitional question, it would return a list of target answers with different levels of confidence in addition to supporting abstracts. If a question was not understood, it would just return abstracts that contained the query terms without the list of possible answers. Thus, while long, complex questions tended to lead to no results from EAGLi and HONQA, askHEREMES returned results for any size and type of question posed. This strategy strongly suggests itself as a general architectural feature for future QA systems.

## Conclusions

There are considerable interesting differences between the three systems. HONQA returns single-sentence answers that are clear and easy to understand. Although EAGLi provides single entity answers, it still seems to be often necessary to read the abstracts to validate the answers provided. It also presents the user with many different ratings and views which can be confusing. With its quick responses, askHERMES is currently the most useable of the three systems, especially if it is necessary to make multiple queries. Table 1 summarizes the dimensions and comparisons of the different systems. Biomedical question answering systems are improving and will be ready for prime time, perhaps surprisingly soon. These three systems demonstrate that they are close to becoming valuable tools for the clinical and biomedical fields.

**Table 1:** Question answering system comparison matrix of features for HONQA, askHERMES, and the EAGLi systems

***QA Comparison Matrix***			
	**EAGLi**	**askHERMES**	**HONQA**
Web address	eagl.unige.ch/EAGLi	http://www.askhermes.org	services.hon.ch/cgi-bin/QA10/qa.pl
Data sources	MEDLINE abstracts	MEDLINE abstracts, eMedicine, clinical guidelines, PubMedCentral, and Wikipedia	HON certified websites
Answers	Multi-phrase passages and a list of single entities	Multiple sentence passages	Sentence
Language	English	English	English/French
System response	Slow	Fast	Slow
Interface complexity	Complex but many tooltips	Simple	Very simple
Question analysis	Yes	Yes	Yes
Target question types	Definition	All types	Definition, procedure, factoid, who
Key feature	Returns a list of ranked terms to answer ‘‘factual‘’ questions	Answers are presented in three ways: answers clustered by terms, simple ranked answer list, and answers clustered by content	Use of certified health websites which allow for information to be geared towards people with varying levels of health literacy

## Competing interests

The authors declare that they have no competing interests.

## Authors’ contribution

MAB performed the system comparisons and wrote the manuscript which was revised by DB. All authors read and approved the final manuscript.

## References

[B1] Wren JD Question answering systems in biology and medicine - the time is now Bioinformatics 2011 27 14 2025 2026 10.1093/bioinformatics/btr327 21672971

[B2] Zweigenbaum P Question answering in biomedicine 2003 Proceedings of the EACL 2003, Budapest 1 4

[B3] Athenikos SJ Han H Biomedical question answering: a survey Comput Methods Programs Biomed 2010 99 1 1 24 10.1016/j.cmpb.2009.10.003 19913938

[B4] Lee M Cimino J Zhu HR Sable C Shanker V Ely J Beyond information retrieval - medical question answering. AMIA Annual Symp Proc 2006 2006 469 473 PMC1839371 17238385

[B5] Cohen AM Hersh WR A survey of current work in biomedical text mining Brief Bioinform 2005 6 1 57 71 10.1093/bib/6.1.57 15826357

[B6] IBM Watson IBM 2011 http://www-03.ibm.com/innovation/us/watson/

[B7] Cao Y Liu F Simpson P Antieau L Bennett A Cimino JJ Ely J Yu H AskHERMES: an online question answering system for complex clinical questions J Biomed Inform 2011 44 2 277 288 10.1016/j.jbi.2011.01.004 21256977 PMC3433744

[B8] Cao Y Cimino JJ Ely J Yu H Automatically extracting information needs from complex clinical questions J Biomed Inform 2010 43 6 962 971 10.1016/j.jbi.2010.07.007 20670693 PMC2991382

[B9] AskHermes The clinical question answering system 2011 University of Wisconsin-Milwaukee http://www.askhermes.org/

[B10] Gobeill J Tbahriti I Ehrler F Ruch P Vocabulary-driven passage retrieval for question-answering in genomics Proceedings of the 16th text retrieval conference 2007 TREC, National Institute of Standards and Technology (NIST):, Maryland, USA

[B11] EAGLi The EAGL project's biomedical question answering and information retrieval interface 2011 University of Geneva http://eagl.unige.ch/EAGLi/

[B12] Cruchet S Gaudinat A Rindflesch T Boyer C What about trust in the question answering world? 2009 AMIA Annual Symposium, San Francisco, USA

[B13] Cruchet S Gaudinat A Boyer C Supervised approach to recognize question type in a QA system for health Stud Health Technol Inform 2008 136 136) 407 412 18487765

[B14] HONQA Health On the Net Foundation 2011 http://services.hon.ch/cgi-bin/QA10/qa.pl

[B15] MEDLINE National Institutes of Health 2011 www.nlm.nih.gov/databases/databases_medline.html

[B16] eMedicine Medical reference 2011 WebMD http://emedicine.medscape.com

[B17] PubMed Central Homepage National Institutes of Health 2011 http://www.ncbi.nlm.nih.gov/pmc/

[B18] Health On the Net Foundation Health On the Net Foundation 2011 http://www.hon.ch

[B19] National Library of Medicine National Institutes of Health [homepage on the Internet] 2011 National Institutes of Health http://www.nlm.nih.gov

[B20] HONcodeprinciples - quality and trustworthy health information. Health On the Net Foundation 2011 http://www.hon.ch/HONcode/Conduct.html

